# Beneficial effect of laughter therapy on physiological and psychological function in elders

**DOI:** 10.1002/nop2.190

**Published:** 2018-07-18

**Authors:** Yuki Yoshikawa, Etsuko Ohmaki, Hirohisa Kawahata, Yoshihiro Maekawa, Toshio Ogihara, Ryuichi Morishita, Motokuni Aoki

**Affiliations:** ^1^ Graduate School of Health Sciences Morinomiya University of Medical Sciences Suminoe‐ku Japan; ^2^ Department of Clinical Gene Therapy, Graduate School of Medicine Osaka University Suita Japan; ^3^ Present address: Faculty of Nursing Setsunan University Hirakata City Japan

**Keywords:** blood pressure, geriatric depression scale (GDS), laughter therapy, quality of life (QOL), serotonin, SF‐8

## Abstract

**Aim:**

In the present study we investigated the effect of laughter therapy on physiological and psychological function in older people.

**Design:**

An open‐label trial.

**Methods:**

Seventeen older people who regularly attended an elderly day care centre were recruited. Stand‐up comedy as laughter therapy was performed once a week for 4 weeks. Parameters of physiological and psychological function were evaluated before and after laughter therapy.

**Results:**

Laughter therapy intervention resulted in a significant reduction in systolic blood pressure and heart rate, accompanied by a significant increase in plasma concentration of serotonin and a significant decrease in salivary concentration of chromogranin A. Questionnaire surveys of SF‐8, GDS‐15, and Vitality Index demonstrated alleviation of depression and improvement of sociability and activity in older people. Laughter therapy could be expected to become a practical treatment to improve quality of life of older people in an elderly day care centre.

## 1 INTRODUCTION

The world's population has been rapidly ageing in recent decades. The United Nations reported that the population aged 60 or above comprised 12% of the global population in 2015 and is growing at a rate of 3.26% a year. Under this situation, some developed countries are already reaching a super‐ageing society. Because ageing is associated with progressive degenerative changes in not only organ function but also both physiological and psychological function, resulting in a decline in quality of life (QOL), promotion of the physical and mental health of older people to improve QOL is becoming a more important issue in an ageing society. From the viewpoint of controlling the escalating healthcare/medical cost associated with an increasing elderly population, cost‐free and practical treatment is sought, in addition to established conventional therapies. Various strategies for health promotion of older people have already been investigated, among which laughter therapy has been evaluated in many studies and is expected to be effective.

Laughter (or humour) therapy has been focused on as an easily accessible, noninvasive, nonpharmacological treatment and has been performed in various groups of participants and as a part of various programmes all over the world (Arrick & Mayhan, 2010; Averill, 1969; Bennett, Zeller, Rosenberg, & McCann, 2003; Berk, Tan, & Nehlsen‐Cannarella, 1988; Bhagat & Vallance, 1987; Broadley et al., 2005; Bush, Krukowski, Eddy, Janusek, & Mathews, 2012; Cardillo, Kilcoyne, Quyyumi, Cannon, & Panza, 1997; Cha & Hong, 2015; Eriksson, Johansson, Sarabi, & Lind, 2007; Ghiadoni et al., 2000; Ghodsbin, Sharif, Jahanbin, & Sharif, 2015; Gottdiener et al., 2003; Hayashi et al., 2007). It is possible that the pleasurable feeling induced by laughter or humour could reduce stress and anxiety and have an effect on psychological function of patients with dementia or depression. In fact, some previous clinical studies demonstrated that psychotherapeutic intervention of laughter resulted in beneficial effects on stress, dementia, and depression (Arrick & Mayhan, 2010; Averill, 1969; Bennett et al., 2003; Berk et al., 1988). Also, two reports investigating the effect of laughter therapy on community‐dwelling older people demonstrated that laughter therapy significantly increased self‐rated health (Bhagat & Vallance, 1987) and that it had positive effects on depression and insomnia assessed by GDS, SF‐36, and PSQI scores (Broadley et al., 2005). Moreover, another randomized controlled trial showed that laughter therapy significantly improved general health, somatic symptoms, insomnia, and anxiety, although it did not improve social dysfunction and depression (Bush et al., 2012). In addition, laughter therapy is strongly suggested to have effects on physiological and biological functions. Laughter was reported to influence the immune system and to induce natural killer cell (NK) activity, accompanied by reduction in stress (Bennett et al., 2003; Bhagat & Vallance, 1987; Cardillo et al., 1997; Cha & Hong, 2015). Also, it was demonstrated that a 3‐month consecutive programme of laughter and exercise in older people resulted in a significant increase in bone mineral density and a significant decrease in haemoglobin A1c (Bhagat & Vallance, 1987). Moreover, happy laughter, joyful music, and hobbies are suggested to improve endothelial function (Hirosaki et al., 2013). These findings obtained from previous studies suggest that laughter therapy has a beneficial effect on not only psychological function but also physiological function and could be a practical treatment to improve QOL of older people with deterioration of activities of daily living (ADL) and a decline in mental health due to depressive feelings, impaired cognitive function, reduced vitality, and poor social activity.

Here, we investigated the effects of laughter therapy on physiological and psychological function in older people who regularly attended an elderly day care centre because of impaired ADL or cognitive disorder. A unique point of the present study is that the Japanese style of stand‐up comedy performed by professional comedians was employed as stimulation to cause laughter or a pleasant feeling. Stand‐up comedy was used as laughter therapy once a week for 4 weeks in an elderly day care centre. Changes in blood pressure, heart rate, NK activity, plasma concentration of serotonin, and salivary concentration of chromogranin A (CgA) were measured as biological/physiological parameters, in addition to psychological parameters such as the geriatric depression scale, QOL scale, and vitality scale.

## 2 METHODS

### 2.1 Participants** and study design**

Seventeen participants aged 60 years and older were recruited from people who regularly attended an elderly day care centre, “Gashu‐en”, because of impaired motor or cognitive function. Stand‐up comedy was used as laughter therapy and participants with sufficient cognitive function to enjoy and respond to stand‐up comedy were recruited. Stand‐up comedy, which was performed by a professional Japanese comedian from Yoshimoto Kogyo Co., Ltd. (Osaka, Japan), was carried out once a week at a fixed time in the morning in the presence of participants for 30 min and run for four consecutive weeks. The content of stand‐up comedy was different each time and each comedy session was performed by a different comedian. Stand‐up comedy consisted of comical performances that were simple, visual and participatory, so as to be easily understandable, such as comical magic, joking, impersonation, and so on.

### 2.2 Parameters for assessment

Measurements taken one day before the first show and at the same time on the day after the last show included NK activity, plasma concentration of serotonin, blood pressure (BP), and heart rate (HR), to evaluate the effect of laughter therapy on physiological functions. As one participant did not provide a blood sample, blood analysis was performed in the remaining 16 participants. Extracted blood samples (Eriksson et al., 2007) were immediately placed on ice and stored at 4°C until just before measurement. Measurements were performed within a day.

In addition, the effects of laughter therapy on psychological function were evaluated by questionnaire surveys. Health‐related QOL of the participants was investigated using the 8‐item Short Form (SF‐8). The Japanese version of SF‐8, which is a questionnaire survey of self‐rated health, consists of eight items; Physical Function, Physical Roles (limitation of roles due to physical problems), Bodily Pain, General Health, Vitality, Social Function, Emotional Roles (limitation of roles due to emotional problems), and Mental Health (Ko & Youn, 2011). Mental health was also assessed using the Japanese version of the Geriatric Depression Scale 15 (GDS‐15), which consists of 15 questions addressing self‐reported feelings in daily life and various depressive symptoms (Kuru & Kublay, 2017). GDS‐15 is commonly used as a screening tool for depression in older people. A higher total score indicates more severe depressive symptoms. Moreover, daily activities and vitality were evaluated by Vitality Index (Lebowitz, Suh, Diaz, & Emery, 2011; Meguro, Ouchi, Akanuma, Meguro, & Kasai, 2014). This questionnaire is not self‐rated and is completed by the participant's family who live with the participant. It consists of five items; Waking pattern, Communication, Feeding, On and off toilet, and Rehabilitation/Activity. Each item is rated as three grades (Arrick & Mayhan, 2010; Averill, 1969). One participant declined to answer SF–8 and GDS–15; thus, assessment by these two questionnaires was performed in the remaining 16 participants.

### 2.3 Saliva analysis

Whole saliva samples (200 µl) were collected from each participant using a soft syringe immediately before and after the last show. Extracted saliva samples were immediately placed on ice and stored at 4°C until just before measurement. Measurements were performed within a day. Saliva samples were immediately centrifuged at 13,000 rpm for 2 min, followed by storage at −20°C. The concentration of CgA in saliva was measured using an enzyme‐linked immunosorbent assay (ELISA) kit, according to the manufacturer's instructions.

### 2.4 Ethics statement

The study protocol was approved by the Ethics Committee for Clinical Investigation of Morinomiya University of Medical Sciences (Permit Number: 2011–020). The study was performed in compliance with these institutional guidelines. All of the participants and their families gave written informed consent before entering the study, which was conducted in accordance with the Declaration of Helsinki.

### 2.5 Statistical analysis

All numerical values are expressed as mean ± *SD* in the tables. In the figures, values are expressed as mean ± *SEM*. Datasets were analysed by paired *t *test. Differences with *p* < 0.05 were considered statistically significant.

## 3 RESULTS

### 3.1 **Baseline clinical characteristics of **participant**s**

The baseline clinical characteristics of the participants in this study are presented in Table 1. All participants attended a facility for elderly day care three times a week on a regular basis. There were 17 participants (Bennett et al., 2003; Hayashi et al., 2007). The participants’ age was 77.0 ± 9.1 years. As the MMSE score of each participant was 19 or higher (23.9 ± 3.2), participants with normal cognitive function or mild cognitive impairment were included. Five participants had been receiving antihypertensive medication and BP was well controlled in these participants. No participant was prescribed medication for dementia or antidepressant agents, which affect psychological function, or any drugs that act on the immune function.

**Table 1:** Baseline clinical characteristics of subjects

Age	77.0 SD 9.1 years
Male	3/17
MMSE	23.9 SD 3.2
NK activity	28.1 SD 20.2%
Serotonin concentration	124.3 SD 85.9 ng/ml
GDS score	7.0 SD 4.3
Total score of SF‐8	20.4 SD 6.2
SBP	129.3 SD 14.8 mmHg
DBP	71.3 SD 5.2 mmHg
Heart rate	71.0 SD 4.5 min
Medication	
Antihypertensive drugs	5/17
Antidepressant agents	none
Drugs for dementia	none

Note.

*N* = 17. Values are expressed as mean ± *SD*.

MMSE: Mini Mental State Examination; NK activity: natural killer activity; SBP: systolic blood pressure; DBP: diastolic blood pressure

### 3.2 Effects of laughter therapy on physiological function of participants

**Figure 1:**
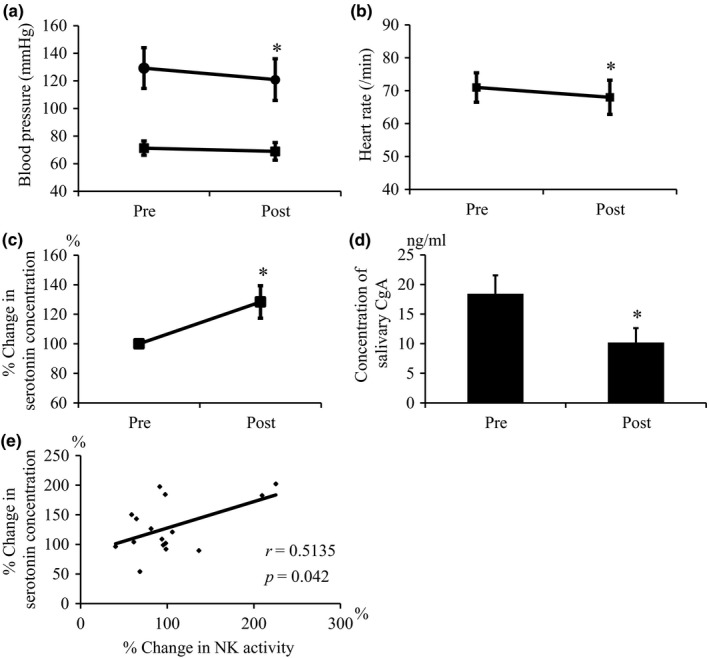
(a) BP before and after intervention. (*N* = 17). (b) HR before and after intervention. (*N* = 17). (c) Percent change in plasma serotonin concentration after four performances of laughter therapy. (*N* = 16). (d) Concentration of salivary CgA before and after laughter therapy. (*N* = 16). (e) Correlation of percent change in plasma serotonin concentration with percent change in plasma NK activity. (*N* = 16). Values are expressed as mean ± *SEM*. ^*^ *p* < 0.05 versus Pre. Pre, the day before the first laughter therapy. Post, the day after the last laughter therapy.

As shown in Figure 1a,b, after repeated laughter therapy for 4 weeks, systolic BP and HR were significantly reduced compared with those before the intervention. In addition, plasma serotonin concentration was significantly increased after repeated laughter therapy compared to that before laughter therapy (Figure 1c). Moreover, a significant decrease in salivary CgA concentration was observed immediately after the last stand‐up comedy show compared to that immediately before the show (Figure 1d). Although there was no significant difference in NK activity between before and after laughter therapy (data not shown), the increase in serotonin concentration by laughter therapy was positively correlated with the increase in NK activity after the intervention (Figure 1e).

### 3.3 Effects of laughter therapy on psychological factors of participants

Questionnaire surveys using SF‐8 and GDS‐15 in this study demonstrated that laughter therapy also significantly improved psychological function. As shown in Table 2, total SF‐8 score was significantly reduced after four interventions of laughter therapy compared to that before intervention. Among the eight items comprising SF‐8, a significant improvement in score was observed for “Bodily Pain” and “Social Function” (Table 2). Also, the GDS‐15 score after four interventions of laughter therapy was significantly decreased in comparison with that before laughter therapy (Table 2). Not only self‐rated scales but also an objective parameter assessed by the participant's family, Vitality Index, was improved. Total Vitality Index score was significantly increased by laughter therapy, as shown in Table 3. Especially, a significant increase in the score of motivation for rehabilitation and other activities was observed (Table 3). All five participants who originally scored 0 or 1 for this item showed improvement.

**Table 2:** SF‐8 and GDS‐15 score

	Pre	Post	*p*
SF‐8 (Total score)	20.44 SD 6.22	15.88 SD 4.84	0.008**
Physical function	3.06 SD 0.68	2.87 SD 0.74	0.255
Physical roles	2.62 SD 1.50	1.60 SD 0.99	0.097
Bodily pain	2.75 SD 1.69	1.67 SD 1.05	0.010*
General health	3.06 SD 0.68	2.87 SD 0.74	0.486
Vitality	2.94 SD 0.68	2.60 SD 0.74	0.136
Social function	2.19 SD 1.42	1.20 SD 0.56	0.034*
Emotional roles	2.13 SD 1.46	1.33 SD 0.62	0.082
Mental health	2.13 SD 1.31	1.33 SD 0.62	0.334
GDS‐15	7.00 SD 4.05	5.63 SD 4.03	0.038*

Note.

*N* = 16. Values are expressed as mean ± *SD*.

*p<0.05 versus Pre (paired *t* test).

**p<0.01 versus Pre.

**Table 3:** Vitality index score

	Pre	Post	*p*
Vitality Index (total score)	9.18 SD 1.38	9.59 SD 1.00	0.030*
Waking pattern	1.94 SD 0.24	1.94 SD 0.24	1
Communication	1.82 SD 0.39	1.94 SD 0.24	0.163
Feeding	2.00 SD 0.00	2.00 SD 0.00	1
On and off toilet	1.76 SD 0.66	1.76 SD 0.66	1
Rehabilitation/activity	1.65 SD 0.61	1.94 SD 0.24	0.020*

Note.

Each item is rated by three grades (2, 1 and 0).

1) Waking pattern

2: Organized pattern of waking, 1: Requires a caregiver’s aid occasionally, 0: Never wakes voluntarily

2) Communication

2: Vocalizes reciprocal exchanges at will, 1: Responsive to verbal stimulation, 0: No cognitive response

3) Feeding

2: Motivated to eat, 1: Passive, but eats with encouragement, 0: Indifferent to eating

4) On and off toilet

2: Independent or never fails to express micturition need, 1: Does not express micturition need consistently, 0: Indifferent to voiding

5) Rehabilitation and other activities

2: Motivated to be rehabilitated or to be involved in other activities, 1: Passive but tries with encouragement, 0: Refuses or indifferent

*N* = 17. Values are expressed as mean ± *SD*.

*p<0.05 versus Pre (paired *t* test).

## 4 DISCUSSION

With the increase in number of older people in an aged society, it is becoming critically important to develop new strategies to improve QOL in older people with impaired physical and/or cognitive function. Day care centres for older people are popular institutions to care for these people, where they regularly attend to receive rehabilitation, food, bathing, and recreation. This study was designed to investigate the effects of laughter therapy as recreation performed in an elderly day care centre and examine the possibility of establishing an easily accessible, noninvasive, nonpharmacological, cost‐free, and practical treatment to improve QOL in older people. No previous report has evaluated the effect of laughter therapy in older people in an elderly day care centre.

The present study demonstrated that laughter therapy significantly improved some physiological functions. One important finding of this study was a significant reduction in BP and HR by regular laughter intervention (Figure 1a,b). This reduction is considered to be induced by parasympathetic activation followed by relaxation and reduced stress by laughter therapy, because early reports demonstrated that humorous stimulation by a funny movie or happy laughter had a beneficial influence on physiological responses determined by BP and HR via autonomic responses (Minami et al., 2015; Nezu, Nezu, & Blissett, 1988). Reduced stress by laughter therapy was also confirmed by lowered levels of stress hormones in this study. The concentration of salivary CgA, which is a sensitive stress hormone that responds in a short time period, significantly decreased immediately after seeing stand‐up comedy, compared to that before the show (Figure 1d). Moreover, laughter therapy for four consecutive weeks resulted in a significant increase in plasma concentration of serotonin, which is known to maintain a stable mental state (Figure 1c), similarly to a previous report (Ghodsbin et al., 2015). Thus, lowering BP and HR by laughter therapy may be followed by suppression of sympathetic nervous system activity via changes in such hormones. Also, the successful reduction of a stress hormone and induction of serotonin indicated that stand‐up comedy could be a useful tool as a method of laughter therapy.

Mental stress is known to affect endothelial function (Hirosaki et al., 2013; Ryu, Shin, & Yang, 2015; Sanchez et al., 2007; Takahashi et al., 2001; Takeda et al., 2010; Toba et al., 2002). It was reported that psychological stress induced by performing an arithmetic or speech task inhibited forearm flow response, indicating endothelial dysfunction (Takahashi et al., 2001; Takeda et al., 2010; Toba et al., 2002). Previous animal experiments partially elucidated its mechanisms, demonstrating that stress hormones, such as glucocorticoids, endothelin‐1, and inflammatory cytokines, caused endothelial dysfunction through the impairment of NO‐dependent vasodilation (Ryu et al., 2015; Sanchez et al., 2007; Toda & Nakanishi‐Toda, 2011; Tokuda et al., 2009). A continuous reduction in stress by laughter therapy may improve endothelial dysfunction in older people via a reduction in glucocorticoids, as previous studies showed that serum concentration of glucocorticoids was significantly decreased in participants who viewed a humorous video compared to control participants (Eriksson et al., 2007; Ryu et al., 2015; Walter et al., 2007). Laughter therapy may also contribute to preventing stress‐induced cardiovascular events in older people through not only lowering BP and HR but also improving endothelial dysfunction mediated by a reduction in glucocorticoids. In addition, the reduction in the glucocorticoid level by laughter therapy would affect immune function, because glucocorticoids are immunosuppressive (Yovetich, Dale, & Hudak, 1990). The reported increase in NK activity by laughter therapy (Bennett et al., 2003; Cardillo et al., 1997; Cha & Hong, 2015; Ghiadoni et al., 2000) may be mediated by a reduction in glucocorticoids. Although the level of NK activity was not changed by laughter therapy in the present study (data not shown), the increase in serotonin concentration by laughter therapy was positively correlated with the increase in NK activity (Figure 1e), suggesting some effects of laughter therapy on immune function.

In most older people, psychological factors are directly connected to social activity and loss of QOL, rather than impairment of physical function. The present study strongly suggests beneficial effects of laughter therapy on psychological function and mental health. The results of depressive feelings in older people in this study were similar to those in previous reports (Broadley et al., 2005). Our finding that GDS‐15 score was improved by laughter therapy once a week for 4 weeks from 7.00 ± 4.05 to 5.63 ± 4.03 (*p* = 0.038) (Table 2) is consistent with previous data showing that laughter therapy at the same frequency changed the score from 7.98 ± 3.58 to 6.94 ± 3.19 (Broadley et al., 2005). The baseline score of participants and the degree of improvement in score were similar in both studies, suggesting that the effect is reliable and that laughter therapy would be useful in older people with a depressive tendency. Moreover, the questionnaire survey of SF‐8 in this study showed a significant improvement in the total score of SF‐8 (Table 2). It is noteworthy that the score of “Social Function” was significantly increased. These data suggest that laughter therapy would improve sociability, preventing social isolation and leading to stronger social ties. Also, the score of “Bodily Pain” was significantly improved in the present study, as well as in a previous report showing an improvement in the score of Bodily Pain in SF‐36 (Broadley et al., 2005). Improved sociability and pain reduction would contribute to improvement of QOL, indicating the usefulness of laughter therapy. Of importance, in the present study, the effects of laughter therapy on psychological function were firstly assessed by the Vitality Index (Table 3), which is not a self‐rated but an objective scale. An interesting finding was the increase in score of motivation for rehabilitation and other activities. These data obtained by questionnaire surveys suggest beneficial effects of laughter therapy on mental health and QOL through an improvement of depressive feelings, sociability, and activity. In addition, a significant increase in serum concentration of serotonin by laughter therapy was observed in this study (Figure 1c). Serotonin is known to act as a neurotransmitter in the central nervous system and to play a major role as a controller of feelings, anxiety, sleep, and vitality. As serum serotonin concentration could reflect intracerebral secretion of serotonin, the effects of laughter therapy observed in the present study might be mediated by induction of serotonin.

Although this study successfully demonstrated some beneficial effect of laughter therapy, its mechanism is still unclear. However, considering the results obtained in this study and previous reports (Eriksson et al., 2007; Ghodsbin et al., 2015; Ryu et al., 2015; Walter et al., 2007), conceivable mechanisms are that changes in physiological parameters are mediated by reduction in stress hormones, such as CgA and glucocorticoids, and that improvement in psychological function is mediated by an increase in serotonin. We observed a reduction in the concentration of salivary CgA immediately after stand‐up comedy and an increase in plasma serotonin concentration after four laughter therapy interventions. However, there are some points that should be considered study limitations. CgA was measured before and after just one show and reproducibility was not confirmed in this study. Also, change in glucocorticoids was not measured in the present study, although a previous study reported that serum concentration of glucocorticoids was significantly decreased in participants who received a laughter therapy programme. In addition, plasma concentration of serotonin was measured in this study, whereas intracerebral secretion of serotonin was not. Moreover, the present study did not reveal how long the effects are maintained. Biological parameters, such as CgA and serotonin, physiological, and psychological parameters should be evaluated in the long term after laughter therapy in a further study.

This study has a few other limitations which should be investigated in further studies. First, the sample size was relatively small. Second, there was no control group and this study did not have a blinded design. Thus, a certain level of bias could not be excluded, especially in self‐rated data. Further randomized, blinded studies using a large number of participants are needed to improve the reliability. Third, despite the importance of a control group, it is difficult to set a rational control group in such studies to clarify the effect of laughter therapy. Although another group without intervention (an untreated group) could be suitable as a control, a different intervention should be considered to be a suitable control. In studies to evaluate laughter therapy, a quantitative parameter to assess the humorousness of various interventions would be needed. Finally, validation of the procedure of laughter therapy was not sufficient in this study. It is still unclear how many times intervention should be provided and for how long it should be continued. The effects of more frequent laughter therapy and an extended period of laughter therapy should be evaluated further. Also, the methodology of causing laughter was not sufficiently evaluated in this study. The method of stimulation of laughter varies among reported studies. In many cases, laughter was forced under guidance as an exercise without a target of a joyful feeling. In contrast, laughter in the present study was caused by the emotional response to viewing and joining in with stand‐up comedy. It is an important point that the stand‐up comedy performed in the present study was designed by professional comedians to be simple, visual, and participatory, so that even older people with somewhat impaired cognitive function could easily understand it and join in. Our method to induce the emotion of comfort may have some advantage in relation to serotonin secretion, different from forced laughter or a laughing exercise as carried out in previous reports. However, no previous study has established a procedure for laughter therapy and the present study did not assess the methodology of laughter therapy.

The type of intervention can be expected to be important. Although there are various types of intervention for laughter therapy, such as a humorous video, slapstick comedy etc., it is still unclear which type is preferable in older people, especially in those with impaired cognitive function. Studies thus far, including the present study, have failed to evaluate what kind of intervention is suitable. Although appreciation of the “sense of humor” of participants receiving various interventions and measurement of the humorousness of each intervention in these studies would help to reveal what kind of intervention is suitable as laughter therapy and to set a rational control group, it is quite difficult to measure and evaluate them. This point is a study limitation and a future task. In addition, when considering a programme of laughter therapy, there may be a difference between spontaneous humour and rehearsed humour. Our programme of laughter therapy using stand‐up comedy is not spontaneous and is more rehearsed humour. In this study, we did not compare the effect of laughter therapy by seeing a show to that of spontaneous humour. As it is difficult to exploit spontaneous humour in participants with impaired cognitive function or a depressive mood, it would be desirable for laughter therapy for older people to be provided as passive humour, where a joyful feeling wells up from seeing a comic show or humorous video.

## 5 CONCLUSION

The present study demonstrated that the intervention of laughter therapy once a week for 4 weeks in an elderly day care centre resulted in a significant reduction in BP/HR, alleviation of geriatric depression/bodily pain and improvement of sociability/activity, accompanied by a significant decrease in salivary CgA level and a significant increase in serum serotonin level. These data strongly suggest beneficial effects of laughter therapy on physiological and psychological functions, although laughter did not affect cognitive function. The present study supports the therapeutic advantage of laughter therapy and raises the opportunity of a new approach to promote physical and mental health in older people. In an aged society, where older people with impairment of ADL and QOL are increasing, laughter therapy could be expected to be a low‐cost, safe, and practical treatment that nurses can use.

## CONFLICT OF INTEREST

The authors declare no conflict of interest.

## AUTHOR CONTRIBUTIONS

YY, RM, and MA: Study concept and design. YY, EO, and MA: Acquisition of participants and/or data. YY, HK, YM, TO, RM, and MA: Analysis and interpretation of data. TO, RM, and MA: Preparation of manuscript.

All authors have agreed on the final version and meet at least one of the following criteria [recommended by the ICMJE (https://www.icmje.org/recommendations/ )]:

- substantial contributions to conception and design, acquisition of data, or analysis and interpretation of data;
- drafting the article or revising it critically for important intellectual content.
